# Nonionic Fast-Penetration System for Diffusion-Driven Degradation of Liquid Plugs

**DOI:** 10.3390/polym17131757

**Published:** 2025-06-25

**Authors:** Yuexin Tian, Yintao Liu, Haifeng Dong, Xiangjun Liu, Jinjun Huang

**Affiliations:** 1 Petroleum Engineering Technology Institute of Southwest Petroleum Branch, SINOPEC, Deyang 618000, China; liuyintao.xnyq@sinopec.com (Y.L.); donghaifeng.xnyq@sinopec.com (H.D.); 2 State Key Laboratory of Oil and Gas Reservoir Geology and Exploitation, Southwest Petroleum University, Chengdu 610500, China; liuxiangjun@swpu.edu.cn (X.L.); huangjinjun@swpu.edu.cn (J.H.)

**Keywords:** liquid plug, nonionic fast-penetration mechanism, structural response behavior, micro-scale mechanical evolution, coupled diffusion–degradation model

## Abstract

Degradable liquid gel plugs are increasingly required for zonal isolation in high-temperature reservoirs, yet their practical deployment is limited by slow internal degradation and insufficient structural failure under diffusive conditions. In this study, a diffusion-driven degradation strategy was developed based on γ-valerolactone and a nonionic fast-penetration agent (Tb), aiming to construct internal pathways and enhance decomposability of a model E51 epoxy–anhydride liquid plug. A multiscale characterization framework, including swelling index evaluation, SEM–EDS, FTIR mapping, CLSM imaging, μ-CT, AFM, and nanoindentation, was applied to investigate degradation behavior under varying temperatures (120–140 °C) and solvent-to-plug ratios (1:1–5:1). The plug exhibited a swelling index of 1.81 in GVL and formed tree-like degradation channels with widths of 20–30 μm. Functional group mapping revealed preferential cleavage of ester and ether bonds at the surface, and mechanical softening (modulus reduction > 57%) was confirmed by AFM and nanoindentation. Higher temperatures and solvent ratios synergistically reduced full degradation time from 84 h to 12 h. These findings validate a “penetration-induced softening–ester bond scission–diffusion channel construction” mechanism, offering an effective design pathway for intelligent degradation control in high-temperature downhole environments.

## 1. Introduction

With the progressive development of deep and unconventional gas reservoirs, the complexity of downhole environments has significantly increased, posing dual demands on sealing materials for high responsiveness and controllable unsealing capability. Liquid plugs, as degradable sealing materials, have been applied extensively in temporary plugging, water control, and assisted gas production in low-pressure fractured reservoirs due to their ease of injection, high sealing strength, and degradability [1,2,3]^.^ Particularly in operations requiring post-treatment channel recovery or multistage cyclic stimulation, the controllability of plug degradation has become a critical index for evaluating field applicability [4,5,6].

However, existing epoxy- or polyester-based plug systems commonly suffer from two technical bottlenecks under high-temperature and high-pressure coupled conditions: (1) limited degradation rate and restricted diffusion pathways, which hinder effective disintegration from surface swelling to internal rupture, as widely reported in recent works focusing on epoxy–anhydride or gel-based plug systems [7,8,9] and (2) the absence of responsive feedback mechanisms during degradation, which can result in incomplete unsealing or delayed breakdown [10,11]. These challenges have posed significant limitations on their field-scale deployment under dynamic downhole environments. Therefore, constructing a new degradable mechanism with accurate responsiveness, controllability, and structural decomposability is essential for next-generation liquid plugging materials.

To address this challenge, considerable efforts have been made globally toward the formulation, diffusion behavior, and degradation mechanism of degradable liquid plugs. These can be categorized generally into three main research directions:

(1) Structural optimization for responsive degradability: Traditional epoxy–anhydride systems offer strong initial sealing performance, yet their high crosslink density imposes rigidity that limits dynamic responsiveness during degradation [12,13,14]. Ding et al. [15] introduced polyester-type degradable monomers to enhance thermal sensitivity and tunability of the degradation window. Similarly, Qin et al. [16] incorporated multifunctional groups into polyester networks to broaden the application window under various downhole conditions. Despite these developments, the degradation response often remains passive and lacks internal dynamic restructuring.
(2) Diffusion-pathway construction and structural response characterization: Diffusion plays a dominant role in controlling degradability. Ren et al. [17] developed a porous plug system that significantly improved the internal diffusion flux of degradation agents. Yao et al. [18] revealed that microcrack-induced primary–secondary diffusion networks could promote a transformation from point-based to area-wide degradation pathways. However, few studies have linked these diffusion pathways directly with the mechanical failure behaviors or structural collapse mechanisms.
(3) Molecular-scale degradation mechanism and reactive interface tracking: Li et al. [19] applied FTIR and SEM to preliminarily correlate ester bond scission with interfacial relaxation. Liu et al. [20] further proposed a combined FTIR mapping–Raman mapping strategy to visualize functional group migration during degradation. Kong et al. [21] used nanoindentation and AFM to establish a correlation between localized elastic modulus reduction and diffusion front propagation, offering a novel perspective on interfacial softening behavior. Tran and Zhou [22,23] built a microfluidic unsealing simulation platform and mapped thermally driven diffusion-assisted degradation mechanisms to verify process coupling in engineering-relevant scenarios.

Despite these advancements, three key scientific gaps remain in the literature support:

(1) The synergistic mechanism of “fast-penetration–degradation” remains poorly resolved, with most studies focusing on macroscopic mass loss or swelling indices without addressing the coupled degradation pathway in depth [10,24].
(2) The causal link between diffusion processes and microstructural failure remains insufficiently resolved in current studies. Existing research has largely focused on evaluating the macroscopic plugging effectiveness or degradation behavior of polymer gels [2,3,4], yet few attempts have been made to systematically correlate the internal diffusion pathway with the initiation and propagation of structural damage.
(3) Interface behavior and reaction pathway coupling in nonionic diffusion systems remain largely unexplored in the existing literature; most related work has focused on nonionic surfactant adsorption kinetics or interface-mediated diffusion behaviors without integrating chemical bond cleavage or structural channel formation into a unified degradation model [25,26,27].

To bridge these gaps, this study proposes a diffusion-assisted degradation strategy based on γ-valerolactone and a nonionic fast-penetration agent using a field-validated E51 epoxy–anhydride liquid plug as a model system. Multidimensional characterization techniques, including SEM/EDS, CLSM imaging, μ-CT reconstruction, FTIR mapping, AFM modulus mapping, and nanoindentation, were employed to construct, for the first time, a mechanism map of “nonionic penetration–swelling softening–structural rupture.” The evolution of functional groups, diffusion pathways, and interfacial softening behaviors was analyzed from molecular to structural scales. Moreover, the coupled effects of temperature and reagent ratio on degradation efficiency and interfacial response were elucidated, offering fundamental guidance for high-performance degradable plug design and staged unsealing control in complex reservoir environments.

## 2. Materials and Methods

### 2.1. Materials

E51 epoxy resin (industrial grade, Runxiang Chemical Co., Ltd., Changzhou, China); epoxidized soybean oil (analytical grade, Kemio Chemical Reagent Co., Ltd., Tianjin, China); methylhexahydrophthalic anhydride (AR grade, Runxiang Chemical Co., Ltd., Changzhou, China); n-butyl glycidyl ether (industrial grade, Runxiang Chemical Co., Ltd., Changzhou, China); octadecyltrichlorosilane (AR grade, Mairui Chemical Technology Co., Ltd., Shanghai, China); γ-valerolactone (GVL, AR grade, Aladdin Reagent Co., Ltd., Shanghai, China); N,N-dimethylacetamide (DMAc, AR grade, Yihua Fine Chemicals Co., Ltd., Beijing, China); N,N-dimethylformamide (DMF, AR grade, Kelon Chemical Reagent Plant, Chengdu, China); heavy aromatic solvents A and B (AR grade, Anpel Scientific Instruments Co., Ltd., Shanghai, China); and proprietary nonionic penetration agents Tb (industrial grade, Sanda Chemical Co., Ltd., Nantong, China) were used.

### 2.2. Instruments

C3003 analytical balance (Wante Balance Co., Ltd., Hangzhou, China), HH-SJ digital thermostatic magnetic oil bath (Guoyu Instruments Co., Ltd., Changzhou, China), DJ1C-60 high-torque stirrer (Jincheng Guosheng Instruments Co., Ltd., Yangzhou, China), DFG101-3 drying oven (Huyue Instrument Co., Ltd., Shaoxing, China), DQ-IV high-temperature/high-pressure core displacement system (Hua’an Scientific Instruments Co., Ltd., Hua’an, China), and MK-LH6 curing reactor (Meike Instruments Co., Ltd., Zibo, China) were used.

Microstructural and chemical analyses were conducted using a ZEISS Sigma 300 field emission scanning electron microscope (Carl Zeiss AG, Oberkochen, Germany) equipped with an Oxford X-act energy-dispersive X-ray spectroscopy module (Oxford Instruments, Abingdon, UK), operated at an acceleration voltage of 10–15 kV. Confocal laser scanning microscopy (CLSM) was performed using a Leica TCS SP8 system (Leica Microsystems, Wetzlar, Germany) with a 488 nm excitation laser. Three-dimensional imaging of internal structures was conducted using a Bruker SkyScan 1272 micro-computed tomography (μCT) scanner (Bruker Corporation, Kontich, Belgium) with a spatial resolution of 0.7 μm, operated at 60 kV and 167 μA.

### 2.3. Preparation of the Liquid Plug

The liquid plug system was formulated using E51 epoxy resin as the base matrix, methylhexahydrophthalic anhydride as the curing agent, n-butyl glycidyl ether as the diluent, and epoxidized soybean oil as the toughening agent. The component mass ratios were as follows: 39.2 wt% epoxy resin, 35.3 wt% curing agent, 13.7 wt% diluent, and 11.8 wt% toughening agent. Under thermostatic stirring at 60 °C, epoxy resin was first combined with epoxidized soybean oil and stirred for 20 min, followed by sequential addition of the diluent and curing agent, and then further stirred for 30 min to obtain a homogeneous, transparent mixture. No catalyst was added to avoid premature initiation; the system underwent thermal curing at 120–140 °C, with the curing time adjustable within 110 h to meet varying crosslink density requirements for subsequent degradation experiments.

### 2.4. Formulation of the Nonionic Penetrating Degradation Solution

**Figure 1:**
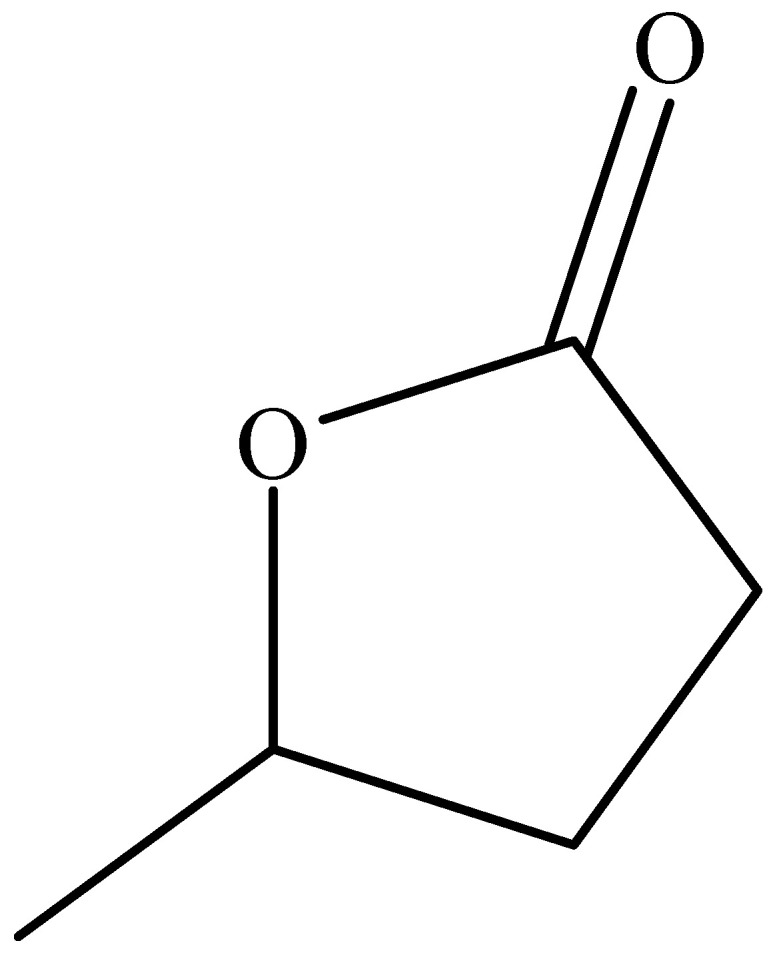
Molecular structure of γ-valerolactone (GVL). GVL is a five-membered cyclic ester (lactone) that exhibits moderate polarity and strong solvent–polymer affinity, making it suitable for swelling and bond cleavage in epoxy–anhydride networks.

A nonionic fast-penetration degradation system was developed comprising (1) an organic solvent with strong swelling ability to loosen the polymer network and initiate structural softening and (2) a nonionic penetrating agent capable of diffusing into the plug and constructing internal transport channels to accelerate molecular-level interactions. GVL and agent Tb were mixed at designated mass ratios and stirred magnetically at room temperature for 30 min to form a homogeneous solution. The degradation tests were conducted with degradation liquid-to-plug mass ratios of 5:1 or 4:1 at 120–140 °C to evaluate the effects of concentration and temperature on diffusion dynamics and interfacial reactivity. The chemical structure of γ-valerolactone (GVL), a five-membered lactone with moderate polarity and high polymer affinity, is presented in Figure 1 to better illustrate its swelling and degradation potential.

### 2.5. SEM and EDS Characterization

Plug specimens were thermally cured, cryogenically fractured, and sputter-coated with gold after vacuum drying to ensure imaging stability. SEM imaging was performed at an accelerating voltage of 5 kV with a working distance below 10 mm and magnifications ranging from 500× to 2000×. EDS was conducted using both point and line scans to examine changes in C and O element distribution before and after degradation to visualize structural evolution and channel development.

### 2.6. Visualization of Penetration Channels

To directly visualize the diffusion behavior of nonionic agents within the plug, a confocal laser scanning microscope (CLSM) was used. A fluorescent dye (Nile Red, 0.05 wt%) was used to label agent Tb and blended with GVL to form the degradation solution. This lipophilic dye exhibits strong fluorescence upon association with the nonpolar structure of Tb, allowing its penetration behavior to be visualized under CLSM. After immersion at 120 °C for 24 h, samples were longitudinally sliced, washed with isopropanol, and dried. CLSM imaging was conducted at an excitation wavelength of 488 nm within 0–100 μm depth from the surface. Images included bright-field, fluorescent, and merged channels to delineate diffusion pathways and interfacial continuity.

### 2.7. FTIR Analysis

FTIR spectra were obtained using a Nicolet iS50 spectrometer (Thermo Fisher Scientific Inc., Waltham, MA, USA) to analyze chemical structure changes before and after degradation. Spectra were recorded over the 4000–400 cm^−1^ range, with a resolution of 4 cm^−1^ and 32 scans per sample. Key peaks of interest included those corresponding to C=O, C–O–C, and N–H groups to track ester bond cleavage and structural rearrangements.

### 2.8. FTIR Mapping

FTIR mapping was conducted using a microscope attachment to generate spatially resolved heatmaps of functional group distribution before and after degradation. Absorbance maps were captured at ~1730 cm^−1^ (C=O), 1100 cm^−1^ (C–O–C), and 3430 cm^−1^ (N–H) to assess group migration and spatial degradation gradients across the sample surface.

### 2.9. AFM Modulus Mapping

An atomic force microscope (Dimension Icon, Bruker) operated in PeakForce QNM mode was used for nanoscale mechanical mapping. Scanning was conducted over a 100 μm × 100 μm region with submicron resolution, enabling heatmap visualization of the elastic modulus across the cross-section before and after degradation and identification of surface softening zones induced by agent penetration.

### 2.10. Nanoindentation

Elastic modulus measurements were carried out using a Hysitron TI 980 TriboIndenter. A maximum load of 5000 μN was applied at a rate of 200 μN/s with indentation depths restricted below 2 μm. At least 10 valid data points were acquired per region. Comparative analysis was performed between the center and edge zones to investigate the spatial heterogeneity and mechanical softening associated with the degradation process.

### 2.11. Evaluation of Degradation Behavior

To assess degradation performance, mass retention and degradation duration were measured. Liquid plugs (10 g each) were molded (Φ2.5 cm × 3.0 cm), thermally cured at 120 °C for 24 h, and then immersed in the prepared degradation liquid under controlled temperatures (120, 130, 140 °C) and reagent-to-plug mass ratios (5:1 to 1:1). Degradation times and morphological changes were recorded.

After degradation, residual solids were filtered, dried, and weighed to calculate mass retention using Equation (1):

(1) $$R=(1-\frac{m_{loss}}{m_{0}})\times 100\%$$

where *R* is the mass retention rate, *m*_0_ the initial sample mass (g), and *m_loss_* the degraded mass.

Additionally, degradation processes under varying concentrations of agent Tb (3–20%) were visually documented to compare the diffusion and structural disintegration rates, validating the proposed diffusion–degradation synergy mechanism.

## 3. Results and Discussion

### 3.1. Diffusion–Swelling Behavior and Structural Response of the Liquid Plug

#### 3.1.1. Macroscopic Volume Expansion and Morphological Evolution Under Solvent Action

**Figure 2:**
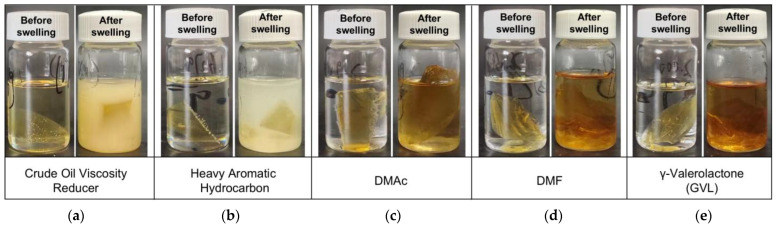
Swelling performance of the liquid plug in various solvents before and after incubation in various solvents at 120 °C for 24 h. ((**a**) Crude oil viscosity reducer. (**b**) Heavy aromatic solvent. (**c**) N,N-Dimethylacetamide. (**d**) N,N-Dimethylformamide. (**e**) γ-Valerolactone).

The initial response of the liquid plug in degradation fluids is typically characterized by the diffusion and penetration of nonionic solvent molecules into the polymer network, leading to notable volume expansion and structural relaxation. In this section, the swelling behavior of the E51-based liquid plug under various solvent environments was comparatively evaluated to reveal its macroscopic expansion characteristics and morphological evolution at an elevated temperature of 120 °C. The swelling states in different solvents are illustrated in Figure 2.

**Figure 3:**
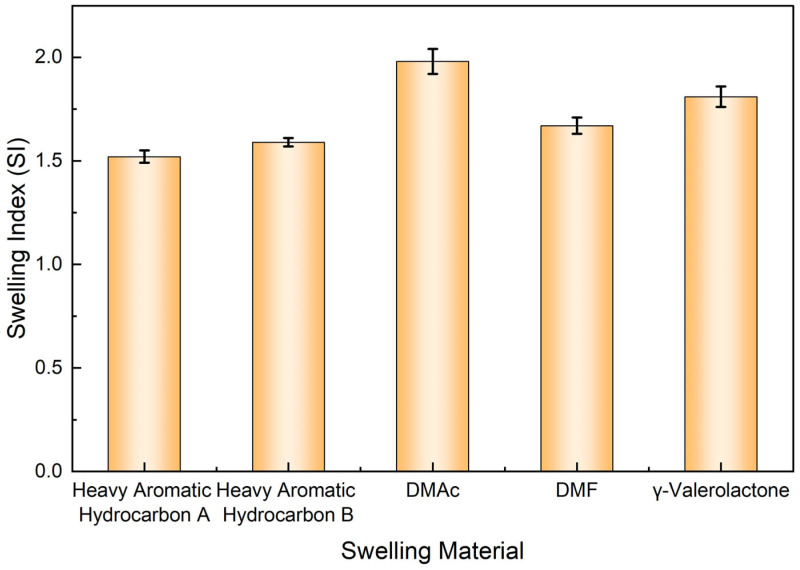
Swelling index of the liquid plug in different solvents.

As shown in Figure 2, the liquid plug treated with N,N-dimethylacetamide (DMAc) maintained an overall intact shape, exhibiting only uniform expansion. In contrast, γ-valerolactone (GVL), N,N-dimethylformamide (DMF), and heavy aromatic solvents induced obvious structural collapse and sheet-like disintegration, suggesting that the solvent type significantly impacts swelling intensity and structural disruption. Although the post-treatment color of the liquid plug in DMF and GVL appears visually similar in Figure 2, their swelling indices and corresponding morphological responses differ significantly, as illustrated in Figure 3. This visual resemblance is likely due to comparable solubilization of residual chromophores or surface oxidation byproducts in both solvents rather than an indication of equivalent swelling performance.

To quantify the swelling effectiveness of each solvent, the swelling index of the liquid plug was calculated and is presented in Figure 3. The swelling index reached a maximum of 1.98 in DMAc, indicating high molecular affinity with the epoxy network. However, no rapid disintegration was observed. By contrast, although the swelling indices of GVL and heavy aromatics were slightly lower (1.81 and 1.59, respectively), local collapse occurred within 3 h, implying a stronger potential to trigger subsequent penetration-induced deconstruction.

To further reveal the thermodynamic mechanism of the swelling process, Hansen solubility parameters (HSP) theory was introduced. According to this theory, the compatibility between solvent and polymer can be judged by the difference in the total solubility parameter δₜ, composed of dispersion, polar, and hydrogen bonding components. The literature reports that the δₜ of E51 epoxy resin is approximately 20.2 MPa^1/2^, while DMAc exhibits a δₜ of 18.5 MPa^1/2^ [28]. The small difference of only 1.7 MPa^1/2^ falls within the highly compatible swelling zone (generally < 2 MPa^1/2^). Thus, DMAc rapidly induces uniform volumetric swelling but lacks sufficient chemical activity to disrupt ester bonds. Similarly, N,N-dimethylformamide (DMF) exhibits a δₜ of approximately 24.8 MPa^1/2^, resulting in a larger mismatch (Δδₜ ≈ 4.6 MPa^1/2^) with the epoxy matrix. Although it has moderate polarity and swelling capability, its low dispersion component and limited chemical reactivity restrict its ability to penetrate deeply or trigger ester bond scission. As such, DMF induces only partial swelling, without causing macroscopic collapse or structural disintegration, making it unsuitable for diffusion-induced degradation applications. In contrast, GVL, with a slightly higher δₜ (~22.1 MPa^1/2^), contains carbonyl and ether groups capable of ester exchange reactions with polyester structures, promoting laminar peeling and network fracture. This “swelling–deconstruction” synergy suggests that solvent screening strategies must balance δ parameter compatibility and structural disruption capability.

In summary, the macroscopic expansion behavior of the liquid plug under various solvents is governed by the dual control of the thermodynamic compatibility and polymer bond responsiveness, following a “volume expansion–structural breakdown” progressive evolution. This provides the necessary physical channel and network loosening foundation for the subsequent penetrant-assisted degradation mechanism. The corresponding microstructural transformations will be further explored in Section 3.1.2 to establish the correlation between macroscopic volume changes and microscopic pore evolution.

#### 3.1.2. Microstructural Evolution Analysis

**Figure 4:**
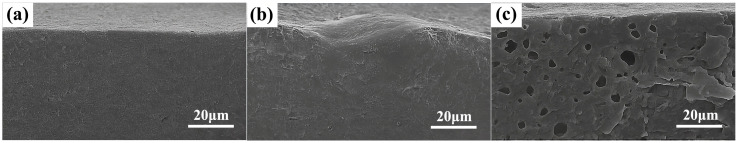
SEM images of liquid plug cross-sections. ((**a**) Untreated, (**b**) treated with DMAc, (**c**) treated with γ-valerolactone. All treated samples were incubated at 120 °C for 24 h before cryo-fracturing and gold sputtering. In (**c**), approximately 34 pores were identified within a 480 μm^2^ field of view, corresponding to a pore density of ~71 pores/mm^2^, indicating significant microstructural loosening).

To elucidate the internal response mechanism of the liquid plug under solvent influence, scanning electron microscopy (SEM) coupled with energy-dispersive X-ray spectroscopy (EDS) was used to characterize the cross-sectional morphology and elemental distribution before and after swelling treatment. The aim was to assess the effect of solvents on network integrity, porosity, and solvent-induced structural disruption. Figure 4 compares the cross-sectional morphologies of untreated, DMAc-treated, and GVL-treated samples.

**Figure 5:**
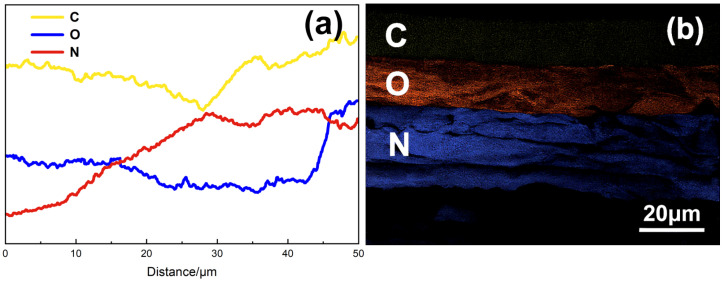
EDS analysis of the liquid plug after γ-valerolactone + Tb treatment at 120 °C for 24 h. ((**a**) Elemental line scanning showing carbon (C), oxygen (O), and nitrogen (N) concentration profiles across the cross-section; (**b**) elemental surface distribution map illustrating vertical stratification of C, O, and N).

As shown in Figure 4a, the untreated liquid plug exhibits a dense structure and smooth interface, indicating high cross-linking and structural integrity. After DMAc treatment (Figure 4b), the internal structure displays slight undulations and micro-expansion but remains continuous, suggesting limited solvent-induced swelling without significant chemical degradation. In contrast, GVL-treated samples (Figure 4c) show prominent lamellar cracking, delamination, and pore formation—typical features of a loosened and porous structure—suggesting that ester exchange-induced chain scission has occurred, creating internal channels favorable for subsequent penetrant diffusion. Quantitative analysis of the SEM image revealed approximately 34 distinguishable pores within the selected region (field of view ~480 μm^2^), corresponding to a pore density of ~71 pores/mm^2^. This data confirms the substantial internal porosity induced by the degradation process and supports the formation of a connected microchannel network. The EDS line scan results in Figure 5a further confirm the composition migration behavior induced by degradation. In the GVL-treated sample, a slight decrease in carbon content was observed near the surface, indicating initial scission of the polyester backbone. A progressive increase in nitrogen (N) content toward the interior was clearly observed, indicating the inward diffusion of nitrogen-containing Tb penetrant groups and their involvement in network disruption. While a modest rise in oxygen (O) was also detected in the 40–50 μm region, this change is likely attributed to the intrinsic oxygen content of γ-valerolactone. Thus, nitrogen migration provides stronger evidence for component penetration and structural modification. These results support the occurrence of a “morphological fracture + compositional reconstruction” dual response, driven by synergistic diffusion–degradation under the GVL + Tb system.

As shown in Figure 5b, the spatial distribution of C, O, and N exhibits clear vertical stratification: surface regions are carbon-dominant, mid-layers are oxygen-enriched, and the inner layers show a nitrogen-rich zone, confirming longitudinal component migration. However, in the EDS line scan results (Figure 5a), the crossover between oxygen and nitrogen around 15 μm and 45 μm may seem inconsistent with the visually distinct boundaries observed in the mapping image. This discrepancy can be attributed to the limited spatial resolution of line scanning, especially for light elements with partially overlapping energy shells, as well as projection effects from inclined channel geometries and local inhomogeneities.

While the 2D elemental mapping provides a spatially averaged visualization with enhanced contrast, the line scan reflects local signal fluctuations along a single trajectory, which may exhibit gradual or diffuse transitions across element boundaries.

Together, these findings further verify that penetrant-induced degradation forms collaborative infiltration pathways and structural collapse zones within the liquid plug. The penetrant forms stress-relief and reactive interface zones within the porous network, facilitating chain scission and structural remodeling—laying the microstructural foundation for the analysis of controlled pathway formation in the next section.

#### 3.1.3. Analysis of Degradation Channel Formation

Efficient degradation of liquid plugs under nonionic penetrant systems depends not only on diffusion kinetics and bond cleavage rates, but also on the development of internal degradation channels. SEM and EDS results previously indicated that the GVL + Tb system induces obvious structural layering and elemental redistribution, implying the formation of continuous infiltration–degradation pathways.

**Figure 6:**
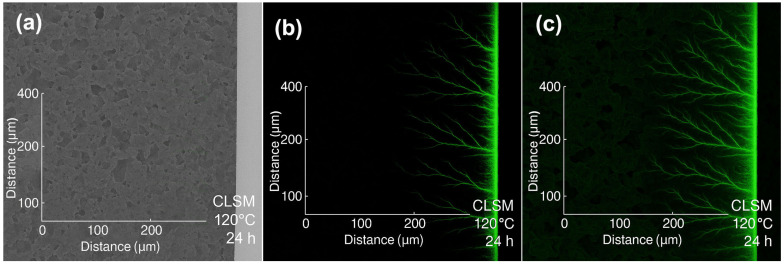
CLSM visualization of dendritic diffusion pathways of the penetrant within the liquid plug after treatment at 120 °C for 24 h. ((**a**) Bright-field image of the untreated control sample (no fluorescence signal; (**b**) CLSM fluorescent image of the sample exposed to γ-valerolactone + 20 wt% Tb, showing strong dendritic diffusion channels extending radially ~400 μm from the surface; (**c**) merged fluorescent and bright-field image confirming channel continuity and internal distribution. The green fluorescence signal originates from Nile red-labeled nonionic penetrant (Tb), highlighting non-uniform diffusion preferentially occurring along weakly cross-linked domains).

To verify this assumption, confocal laser scanning microscopy (CLSM) was employed to trace the diffusion of Tb-labeled penetrant in the liquid plug. The results are shown in Figure 6.

As shown in Figure 6, after treatment at 120 °C for 24 h, the cross-section of the plug displayed well-defined fluorescent channels penetrating radially up to ~400 μm. The dendritic fluorescence pattern suggests non-uniform diffusion preferentially occurring along microcracks or weakly cross-linked regions, forming structurally connected channels. This behavior supports the presence of a structure-driven diffusion mechanism, where penetrants preferentially infiltrate regions of low cross-linking or weak interfacial adhesion to establish reactive fracture zones. These observations agree with the SEM and EDS results, reinforcing the proposed “diffusion–structural response–degradation synergy” mechanism and providing microstructural evidence for subsequent unblocking pathway formation.

Combining Figure 4 and Figure 5, the identified “channel zone” is characterized by (i) connectivity from surface to mid-depth; (ii) O/N element gradients as indicators of active degradation regions; and (iii) spatial overlap with visible surface cracks, suggesting fracture propagation initiates at channel entry points. Based on these findings, a collaborative diffusion–degradation mechanism is proposed: the nonionic penetrant preferentially infiltrates looser polymer regions, promotes ester bond cleavage, and facilitates deeper diffusion of the degradation fluid. This results in the formation of a branched channel network consisting of “primary channels + secondary pathways,” enabling the transition from pointwise swelling to plane-wide structural deconstruction.

This mechanism not only lays the foundation for subsequent unblocking behavior but also offers a theoretical basis for spatiotemporal control of the degradation process, contributing to the design of controllable unsealing windows.

### 3.2. Mechanistic Insights into Diffusion-Driven Degradation Behavior Under Variable Penetrant Concentration

#### 3.2.1. Coupled Effects of Penetrant Concentration on Diffusion Rate and Degradation Kinetics

**Figure 7:**
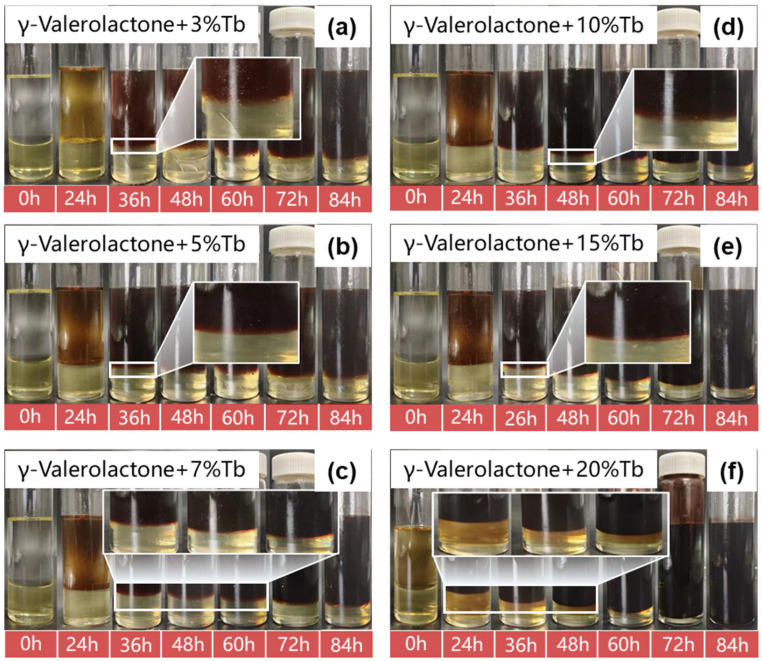
Evolution of degradation behavior of the liquid plug at different Tb dosages ((**a**–**f**) 3% to 20%).

The diffusion behavior of nonionic penetrants within the liquid plug matrix is highly dependent on their concentration, especially under elevated temperature conditions. This concentration not only governs the infiltration rate of solvent clusters but also modulates the overall kinetics of degradation reactions. To elucidate the mechanism of penetrant-assisted degradation, γ-valerolactone (GVL) was selected as the swelling solvent and penetrant Tb was used as a diffusion accelerator. A series of experiments were conducted with various Tb dosages (3%, 5%, 7%, 10%, 15%, and 20% by mass), and the corresponding degradation behaviors of the liquid plug over a period of 0–84 h were recorded (Figure 7). In the experiments presented in Figure 7, the mass ratio of γ-valerolactone to the liquid plug was fixed at 5:1 to ensure consistent solvent conditions across all tested Tb concentrations. In this study, all experiments were conducted at 120 °C to ensure sufficient molecular activity for degradation.

At lower concentrations (3–7%), the liquid plug exhibited only surface softening and peripheral swelling, with no apparent collapse. In contrast, with 10–15% Tb, bulk disintegration and complete separation of the plug matrix were observed, leaving no noticeable residue. At 20% concentration, degradation was significantly accelerated, achieving full dissolution within 84 h.

**Figure 8:**
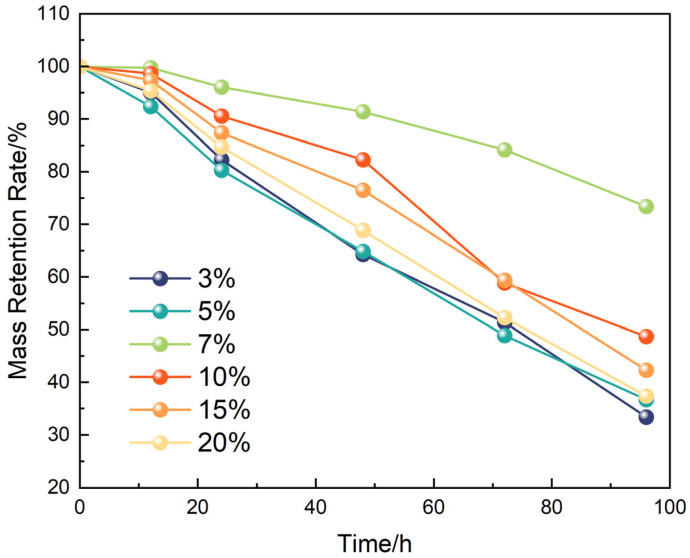
Degradation rate of the liquid plug as a function of Tb concentration.

Quantitative evaluation of the degradation behavior is presented in Figure 8. The mass retention rate at 60 h decreased from ~62% to ~28% as Tb content increased from 3% to 20%, revealing a strong inverse correlation between penetrant concentration and plug stability. This trend suggests that higher Tb dosages greatly enhance non-steady-state diffusion dynamics and internal structural failure.

Mechanistically, the nonionic penetrant Tb facilitated degradation via two synergistic pathways: (1) by reducing interfacial tension to destabilize the polymeric network and (2) by establishing internal channels that enabled uniform expansion of the swelling solvent. The observed fracture morphology under 20% Tb treatment further validated the proposed “diffusion-induced disintegration” mechanism.

In summary, increasing Tb concentration not only boosted degradation kinetics but also transformed the degradation pathway from superficial swelling to in-depth structural disintegration, thereby constructing a diffusion-driven, controllable degradation system. This provides a microstructural basis for modeling degradation mechanisms and constitutive behavior.

#### 3.2.2. Structural Evolution and Channel Formation During Diffusion–Degradation Coupling

**Figure 9:**
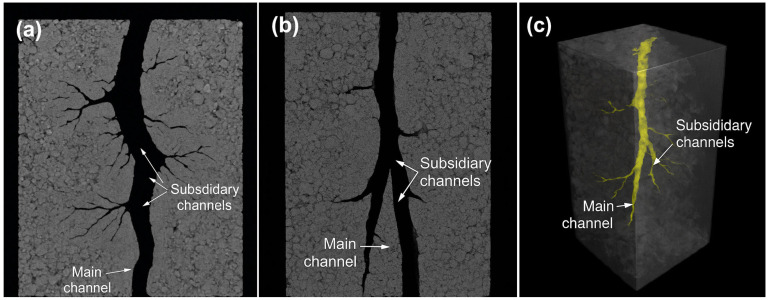
μ-CT reconstruction of channel structures induced by nonionic penetrant. (**a**) Transverse cross-section showing a main channel and numerous radial subsidiary branches forming a sparse peripheral network; (**b**) Longitudinal view displaying asymmetric bifurcations and multiscale channel distributions; (**c**) 3D rendering visualizing a central backbone structure with capillary-like peripheral branches.

While previous SEM and CLSM analyses revealed microstructural disruption and branched diffusion channel formation under penetrant action, these two-dimensional observations could not fully capture the connectivity and spatial topology of degradation pathways. To establish a comprehensive 3D structural model, micro-computed tomography (μ-CT) was employed to perform non-destructive imaging of liquid plugs after degradation in 20 wt% Tb for 84 h. High-resolution scanning (0.7 μm/voxel) was conducted using the SkyScan 1272 system (Figure 9).

Transverse cross-sections (Figure 9a) show a primary channel penetrating the plug radially with numerous secondary branches extending outward, forming a sparse peripheral network. Longitudinal views (Figure 9b) reveal multiscale bifurcations and asymmetric spatial distribution of the channels. The 3D rendering (Figure 9c) clearly visualizes a hierarchical structure of a central backbone and peripheral capillary-like branches.

**Figure 10:**
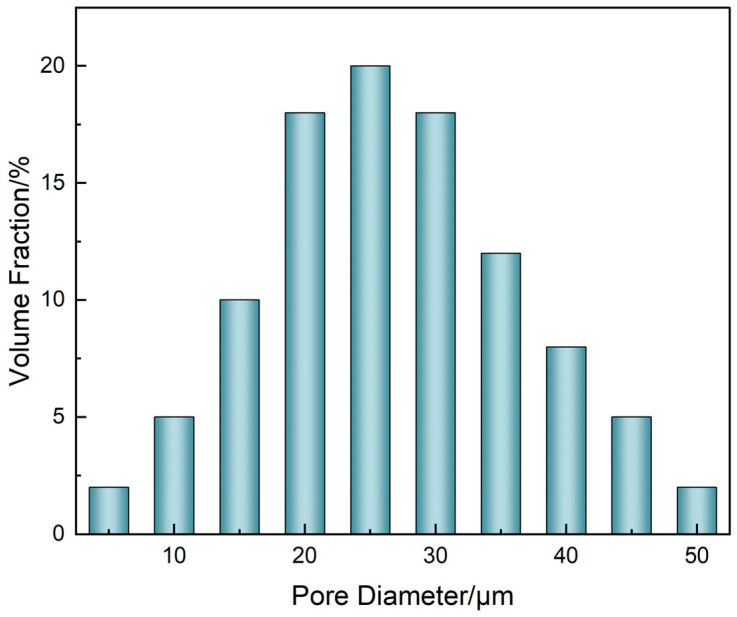
Histogram of degradation channel diameters.

To quantify the structural characteristics of the μ-CT reconstructed network, the following analysis protocol was adopted:

(1) Channel diameters were extracted using ImageJ (v1.54) with the BoneJ plugin, employing the “Thickness” function based on sphere-fitting geometry across over 800 slices, from which the diameter histogram (Figure 10) was constructed.
(2) Connectivity was defined as the ratio of connected pore voxels to total pore voxels within the segmented region, calculated using Bruker CTAn software (version 1.16) based on a 26-voxel neighborhood rule.
(3) Effective permeability was estimated using Avizo XLab Hydro(version 2020.2) via steady-state Navier–Stokes simulation under a pressure gradient of 100 Pa. The segmented μCT domain (resolution 0.7 μm/voxel) was used directly as the simulation input.

These tools enabled the reliable and reproducible quantification of transport-relevant metrics based on the experimentally reconstructed 3D pore structure.

Quantitative analysis of the reconstructed network (Figure 10) indicated that most channels had diameters in the 20–30 μm range, forming a unimodal pore size distribution favorable for continuous degradation.

**Figure 11:**
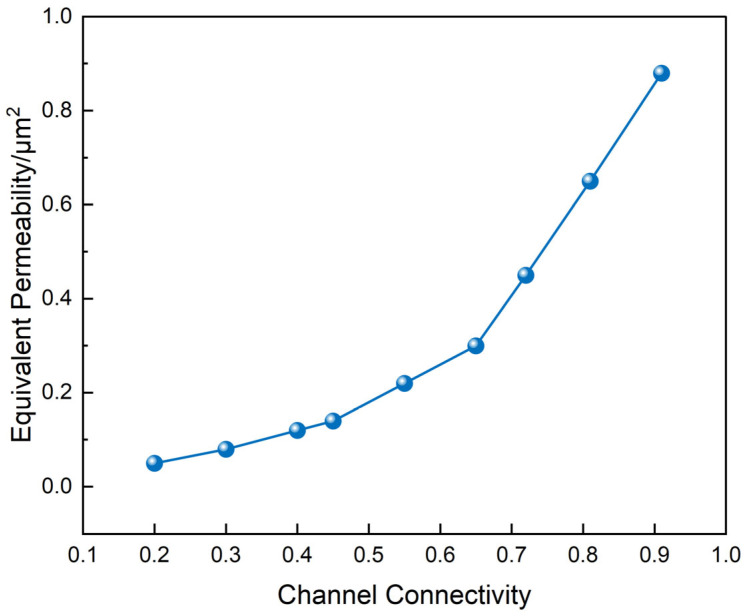
Relationship between connectivity and effective permeability.

A correlation between channel connectivity and effective permeability was established based on the 3D reconstruction (Figure 11). As connectivity increased from 0.2 to 0.95, permeability increased nonlinearly from 0.05 to 0.88 μm^2^. A permeability surge was observed above 0.75 connectivity, suggesting that a fully connected “main branch” diffusion system had been established.

Integration of the μ-CT results across Figure 9, Figure 10 and Figure 11 reveals a coherent structure–dimension–function correlation governing the degradation process. The hierarchical channel network observed in Figure 9, comprising a central main channel and numerous capillary-like branches, directly aligns with the unimodal pore diameter distribution in Figure 10 centered around 25.3 μm. This suggests that both structural levels—primary and subsidiary—fall within the effective diffusion size range. Furthermore, the sharp rise in permeability beyond 0.75 connectivity in Figure 11 corresponds to the stage when subsidiary branches merge with the main network, as visualized in Figure 9c. This supports a percolation-like transition driven by spatially extended dendritic channel formation. Collectively, these findings demonstrate that nonionic penetrant-induced degradation not only alters morphology but also creates a transport-effective architecture with scalable permeability.

These results confirm the three-phase coupling mechanism proposed in Section 3.1 and Section 3.2: (i) selective structure weakening, (ii) path-guided evolution, and (iii) controllable channel formation. The μ-CT platform provided structural verification and quantitative support for modeling degradation–unsealing processes.

Although this study primarily focused on the irreversible degradation behavior, it is worth noting that the observed channel formation under penetrant action is not a reversible process. The penetrant-induced structural evolution involves permanent chemical bond scission (e.g., ester and ether bonds), irreversible mechanical softening, and morphological collapse. These features collectively indicate a unidirectional transformation rather than a reversible swelling–recovery cycle.

#### 3.2.3. Molecular Degradation Pathways and Functional Group Migration Analysis

**Figure 12:**
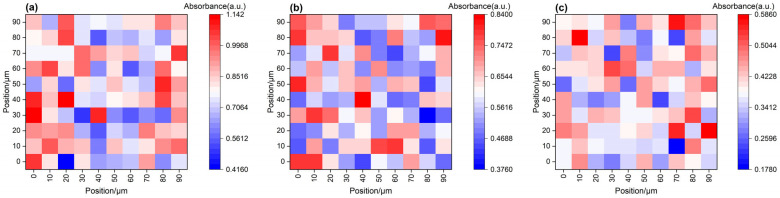
Spatial FTIR mapping of functional group changes before and after degradation. (**a**) C=O stretching (1730 cm^−1^), (**b**) C–O–C asymmetric stretching (1150 cm^−1^), and (**c**) N–H bending (1550 cm^−1^). The color scale represents normalized absorbance (a.u.): red indicates stronger signal intensity; blue indicates weaker or depleted signal. All samples were treated with 20 wt% Tb at 120 °C for 24 h.

To further investigate the degradation pathways, Fourier-transform infrared mapping (FTIR mapping) was conducted to spatially resolve functional group changes in untreated and degraded samples (20 wt% Tb, 120 °C, 24 h). Relative intensity maps for C=O (1730 cm^−1^), C–O–C (1150 cm^−1^), and N–H (1550 cm^−1^) are shown in Figure 12a–c.

The colormap indicates normalized absorbance intensity (from blue to red), where red regions correspond to higher local absorbance and blue indicates depletion. For the C=O band (Figure 12a), surface-localized intensities dominate, suggesting that ester group cleavage occurs preferentially near the exterior. Similarly, C–O–C absorbance (Figure 12b) shows a gradual surface-to-core decay, consistent with ether bond degradation during solvent penetration. In contrast, N–H distribution (Figure 12c) remains spatially uniform, confirming that amine functionalities are chemically stable during degradation.

This FTIR mapping highlights chemical selectivity in the degradation process and spatial localization of bond cleavage, supporting the mechanism of surface-initiated molecular fragmentation driven by penetrant infiltration.

**Figure 13:**
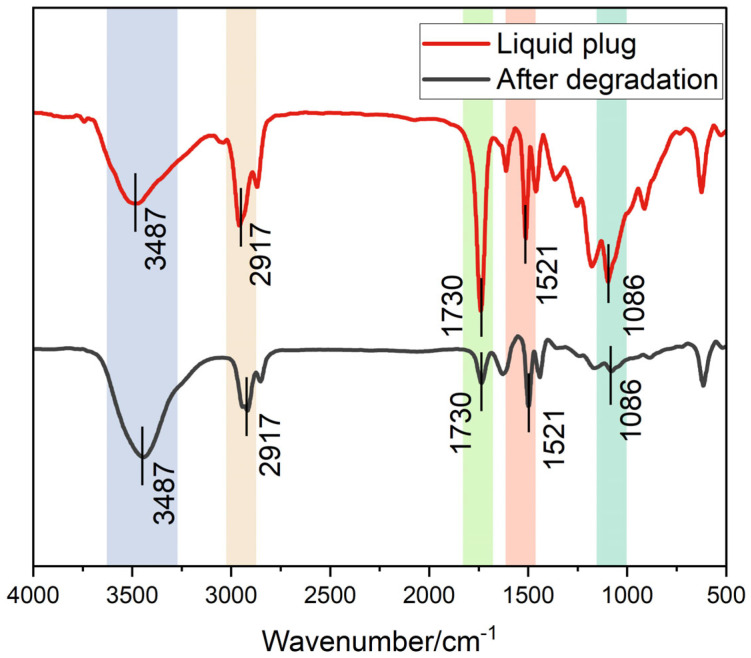
FTIR spectra comparison before and after degradation (Spectra were baseline corrected and intensity normalized to the C–H stretching band at 2917 cm ^−1^. Peak weakening of C=O (1730 cm ^−1^), broadening of C–O–C (1086 cm ^−1^), and enhancement of O–H/N–H (~3487 cm ^−1^) suggest selective cleavage of ester and ether bonds).

The corresponding FTIR spectra before and after degradation are presented in Figure 13. Prior to analysis, all spectra were baseline corrected and intensity normalized with respect to the C–H stretching peak at 2917 cm^−1^, which remained chemically stable during degradation and served as an internal reference.

Post-degradation, the C=O stretching peak at 1730 cm^−1^ significantly weakened, indicating ester bond cleavage, while the broad O–H/N–H absorption at ~3487 cm^−1^ was notably enhanced, suggesting exposure of polar groups due to hydrolysis or network disruption. The C–O–C vibration band near 1086 cm^−1^ exhibited broadening and redshift, supporting scission of ether linkages. These spectral features collectively confirm that GVL/Tb-induced degradation preferentially disrupts ester and ether bonds while leaving the polymer backbone relatively intact, consistent with the selective chemical remodeling observed in FTIR mapping.

These results confirm that degradation under GVL/Tb conditions involves spatially selective bond cleavage, primarily affecting ester and ether functionalities while preserving the main polymer backbone.

#### 3.2.4. Local Mechanical Softening and Interface Weakening Mechanism

**Figure 14:**
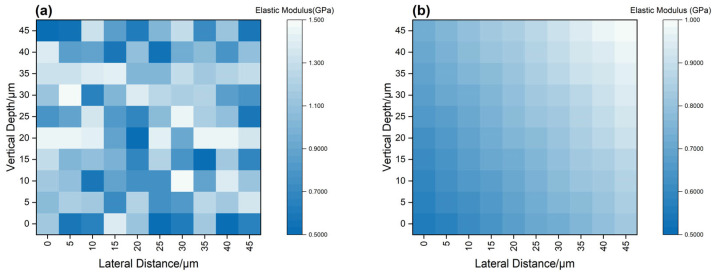
Spatial evolution of elastic modulus before and after degradation: (**a**) AFM modulus map of degraded sample (20 wt% Tb, 120 °C, 24 h) showing a mechanically weakened layer with reduced stiffness; (**b**) reference sample without degradation, exhibiting uniform high modulus). Color scale indicates local elastic modulus in GPa.

To quantify mechanical degradation, nanoindentation and AFM modulus mapping were employed to measure spatially resolved elastic moduli of untreated and degraded samples (Figure 14). The left panel corresponds to the degraded sample after 24 h exposure to 20 wt% Tb at 120 °C, and the right panel represents the untreated control.

Post-degradation, the elastic modulus at the surface decreased markedly from ~1.35 GPa to ~0.58 GPa, forming a mechanically weakened gradient layer extending ~30 μm from the surface. In contrast, the untreated sample exhibited spatial uniformity in modulus (~1.35 GPa), indicating consistent crosslink density throughout the matrix. The observed softening correlates well with the FTIR mapping results in Figure 12, where ester and ether bond cleavage were most pronounced near the surface. This spatially resolved softening behavior also mirrors the CLSM results showing penetrant infiltration pathways extending up to 400 μm.

These findings confirm that degradation initiates from the surface, with progressive softening and crosslink relaxation occurring inward, forming a compliant interfacial layer that facilitates structural disintegration under stress.

**Figure 15:**
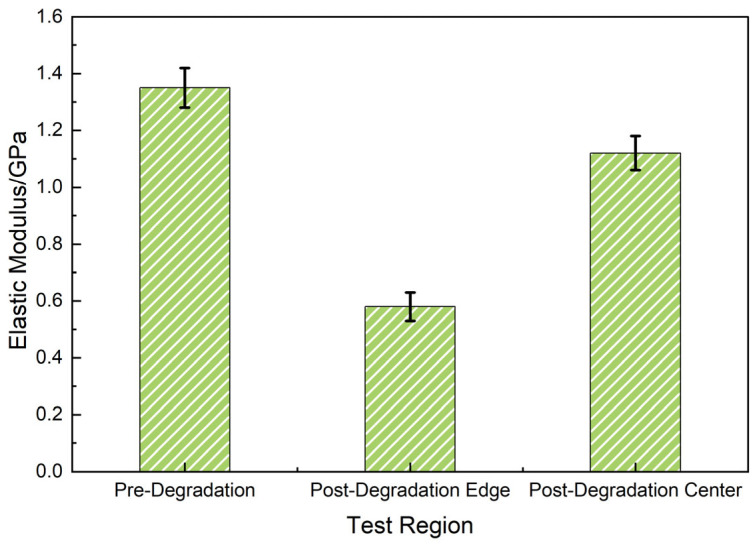
Quantitative comparison of elastic modulus across regions.

Further statistical comparison via nanoindentation (Figure 15) showed minimal variation in untreated samples (center and edge: 1.35 ± 0.07 GPa) but significant softening post-degradation (center: 1.12 ± 0.06 GPa; edge: 0.58 ± 0.05 GPa). These results corroborated the formation of a mechanically weak interface under diffusion-driven degradation.

Collectively, the mechanical data closed the “chemical–structural–diffusion” loop of the degradation mechanism and supported the inclusion of a softening parameter in permeability modeling.

### 3.3. Construction of a Synergistic Penetration–Degradation Mechanism Map and Analysis of Interfacial Regulation Behavior

#### 3.3.1. Mechanistic Imaging and Structural Evolution Under Synergistic Penetration–Degradation Conditions

**Figure 16:**
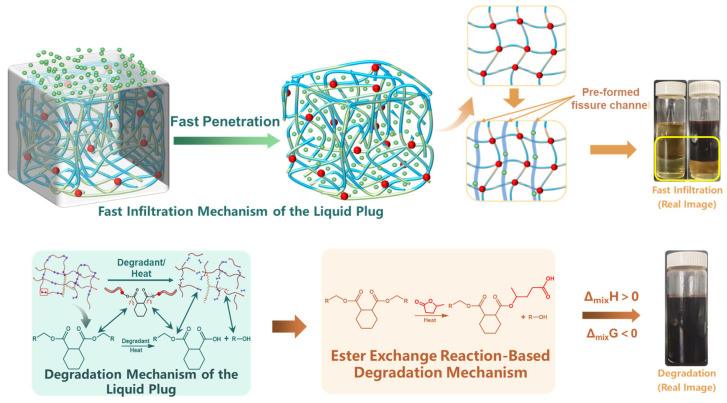
Schematic of the synergistic degradation mechanism for the liquid plug under nonionic penetration conditions (arrows in the figure are used to indicate the direction of the degradation process and do not represent quantitative or mechanistic data).

Based on the preceding investigations into swelling response and penetrant diffusion in the liquid plug, this section integrates the multistage behaviors of the entire degradation process and constructs a comprehensive synergistic mechanism map under the nonionic penetration system, as illustrated in Figure 16.

At the initial stage, nonionic penetrants rapidly diffuse into the surface layer of the liquid plug via intermolecular interactions (top left of Figure 16), forming precursor penetration pathways centered around polar group enrichment zones. This process leads to increased free volume and network relaxation, inducing preferential cleavage of surface functional groups. Subsequently, regions with reduced crosslinking density evolve into microcrack zones, promoting further inward diffusion of the penetrant (top center of Figure 16), ultimately resulting in structural failure in the mid-depth region.

This mechanism is supported by the FTIR mapping results in Figure 12a–c, which show that the C=O and C–O–C absorption bands exhibit a distinct surface enhancement–core attenuation trend, while the N–H bands remain relatively uniform. These patterns confirm that the degradation preferentially targets ester and ether-linked network structures.

Further validation is provided by the infrared spectral evolution in Figure 13, where post-degradation samples show intensified C=O peaks (1720–1750 cm^−1^) and red-shifted, broadened –OH (3430 cm^−1^) and C–O–C (1100 cm^−1^) bands. These spectral changes indicate not only physical diffusion but also molecular-scale ester bond cleavage, crosslink rearrangement, and polar group reconstruction. These transformations correlate with the formation of acid and alcohol degradation byproducts, as shown in the lower part of Figure 16, where γ-valerolactone undergoes ester exchange reactions under thermal penetration conditions, leading to chain scission and fragment release. From a thermodynamic standpoint, this is characterized by Δ_mix_H > 0 and Δ_mix_G < 0, indicating a spontaneous process driven by enthalpic and entropic contributions.

#### 3.3.2. Regulation of Interfacial Response Behavior by Temperature and Degradation Liquid Ratio

**Figure 17:**
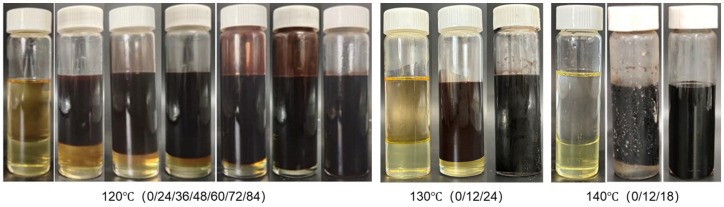
Degradation morphology of liquid plugs at different temperatures.

**Figure 18:**
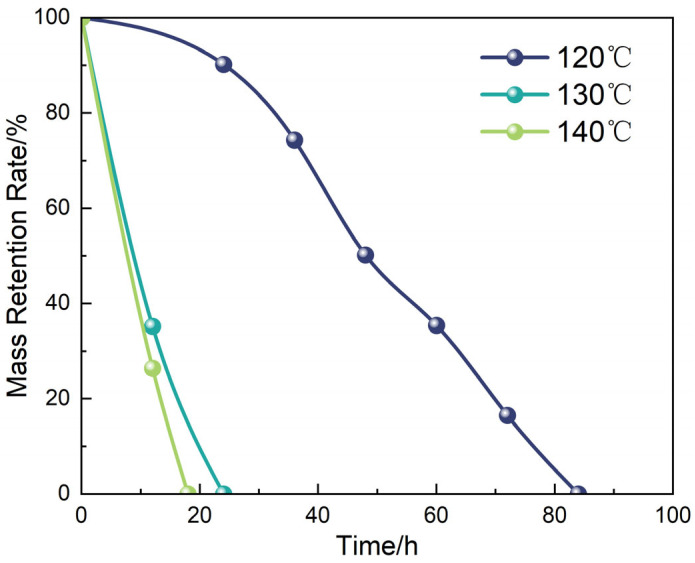
Corresponding degradation rate at varying temperatures.

**Figure 19:**
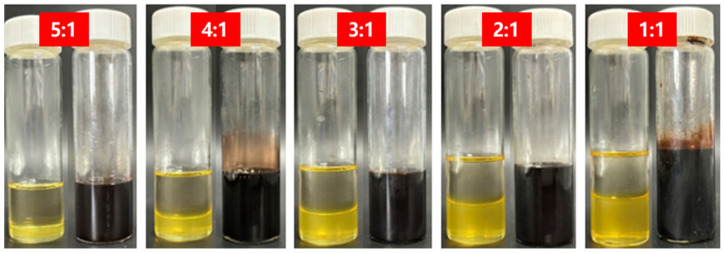
Morphological variation under different degradation fluid ratios. (Photographs show the visual changes in liquid plugs after exposure to degradation fluid with varying mass ratios of GVL + 20 wt% Tb to plug material (from left to right: 5:1, 4:1, 3:1, 2:1, and 1:1). All degradation experiments were conducted at 120 °C for 24 h. With decreasing fluid-to-plug ratios, the degradation extent progressively declined, as indicated by the residual solid volume and turbidity of the surrounding solution).

**Figure 20:**
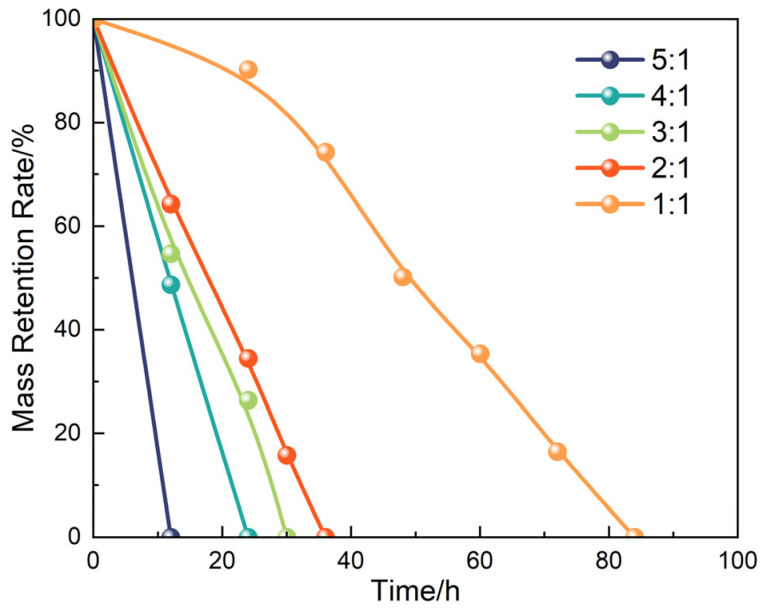
Degradation rate as a function of fluid-to-solid ratio.

Beyond validating the degradation mechanism, the interfacial tunability of the system was further investigated by examining the influence of temperature and liquid-to-solid mass ratio on the degradation behavior of the liquid plug in the GVL + Tb nonionic system. Figure 17 and Figure 18 display morphological changes at different temperatures (120 °C, 130 °C, 140 °C) under a fixed degradation fluid ratio. The tested system was consistently formulated using γ-valerolactone with 20 wt% Tb. Figure 19 and Figure 20 illustrate the effects of different fluid-to-plug mass ratios (5:1 to 1:1) on the degradation behavior.

The experimental results reveal that increasing temperature significantly accelerates both the diffusion rate of the penetrant and the cleavage of the ester bonds. Specifically, the complete degradation time decreased from 84 h at 120 °C to 18 h at 140 °C. Similarly, increasing the liquid-to-plug ratio from 1:1 to 5:1 reduced the full degradation time from 84 h to 12 h. These findings indicate that elevated temperatures enhance molecular mobility and penetration flux, promoting disruption of the plug’s internal network, while a sufficient degradation liquid supply maintains a steep concentration gradient and suppresses the accumulation of byproducts, ensuring continuous interfacial reaction progression.

Together, these effects drive the penetration–degradation process along the material’s structural gradient. This temperature–diffusion coupling mechanism establishes a dual-triggered response model, providing both theoretical and practical guidance for dynamically regulating the sealing–unsealing window in subsurface engineering applications.

## 4. Conclusions

(1) The incorporation of octadecyltrichlorosilane (OTS) as a bifunctional interfacial modifier significantly enhanced the interfacial bonding performance between the liquid plug and steel substrate. Among the tested variants, OTS-18, featuring an 18-carbon alkyl chain, demonstrated the most pronounced strengthening effect at a concentration of 0.25 wt%, increasing bonding strength by 445% and shear strength by 73.8% compared with the unmodified interface. This study systematically revealed the regulatory role of OTS chain length on plug–steel interfacial coupling, providing molecular design parameters for sealing material modification.
(2) Analysis of the interfacial microstructural evolution mechanism showed that OTS molecules constructed a flexible interlayer and root-like interlocking units on the steel surface via a synergistic “anchoring–entanglement–buffering” mechanism. The penetration depth of the interfacial layer increased from 39.6 nm to 391.6 nm, effectively enhancing stress dispersion and crack propagation resistance. This multiscale synergistic pathway validated the OTS-induced interfacial enhancement mechanism of “wetting–penetration–locking,” offering a mechanistic solution to interfacial failure in liquid plug systems.
(3) A structure–property coupling model was established integrating the contact angle, interfacial free energy, penetration depth, and mechanical metrics (shear and bonding strength). This model enabled, for the first time, a full-pathway interpretation from molecular-scale interfacial activation to macroscopic mechanical performance improvement. The findings not only propose an interfacial design methodology suitable for liquid plug systems, but also provide theoretical and practical guidance for the development of high-reliability wellbore sealing materials and interfacial modification strategies in complex downhole environments.
(4) The degradation-induced formation of internal channels was confirmed to be an irreversible process, involving permanent structural disruption and elastic modulus reduction. This supports the unidirectional nature of the penetrant–material interaction and ensures a robust functional transition from sealing to unblocking, thereby establishing a time-controlled degradation pathway for practical field deployment.

## Data Availability

The figures and tables used to support the findings of this study are included in the article.
